# Tomotherapy concomitant with cetuximab, followed by cetuximab as single-agent therapy for unresectable squamous cell carcinoma of the skin: a case report

**DOI:** 10.1186/1471-5945-14-15

**Published:** 2014-09-30

**Authors:** Sara Falivene, Francesca Maria Giugliano, Antonio Maria Grimaldi, Rossella Di Franco, Diego Toledo, Matteo Muto, Fabrizio Cammarota, Valentina Borzillo, Paolo Antonio Ascierto, Paolo Muto

**Affiliations:** 1 Dipartimento di diagnostica per immagini e Radioterapia – Seconda Università degli studi di Napoli, Piazza Miraglia, 80131 Naples, Italy; 2 UOC Radioterapia -– Istituto Nazionale per lo studio e la cura dei tumori “Fondazione Giovanni Pascale” – IRCCS, Via Mariano Semmola 80131 Naples, Italy; 3 SC Oncologia Medica Melanoma Immunoterapia Oncologica e Terapie Innovative – Istituto Nazionale per lo studio e la cura dei tumori “Fondazione Giovanni Pascale” – IRCCS, Via Mariano Semmola 80131 Naples, Italy; 4 EuropeanMedicalImaging - Fondazione Muto-onlus, Napoli, Italia, Via Taverna Rossa, 169, 80020 Casavatore, Naples, Italy

**Keywords:** SCC, Cetuximab, Tomotherapy, Target therapy, Quality of life

## Abstract

**Background:**

Cutaneous squamous cell carcinoma (SCC) is the second most frequency of all skin tumors. Incidence of SCC has risen significantly due to an increased sun exposure and the number of immunodeficient patients. Cutaneous SCC is characterized by high Epidermal growth factor receptor (EGFR) expression with low frequency of RAS mutations. Generally, locoregional surgery is curative and systemic therapy is not indicated. We evaluated the activity and toxicity profile of tomotherapy concomitant with Cetuximab, followed by Cetuximab as single agent therapy in a patient affected by unresectable, locally advanced cutaneous SCC.

**Case presentation:**

At our institution, on March 2012 we treated a 45 years-old patient affected by locally advanced, unresectable G1 SCC of the lumbar region. At our first observation, the patient was asthenic, with severe pain and functional limitations. There was also a superinfection due to Pseudomonas Aeruginosa resistant to antibiotics, and a G3 anemia secondary to the bleeding lesion. ECOG Performance Status was 2. Tomotherapy has been performed concomitant with the Cetuximab (400 mg/m2, followed by weekly doses of 250 mg/m2) at the total dose of 60 Gy (2 Gy/fx), followed by Cetuximab monotherapy.

The lesion reduced progressively until disappear even after the suspension of the treatment and the patient achieved complete response. Toxicity resulted in G1 cutaneous rash and G2 toxicity to the nails, appeared after 5 months of treatment, typical toxicity profile of the anti-EGFR therapies. After one month of therapy the Pseudomonas Aeruginosa superinfection totally disappeared. Quality of life resulted significantly improved with reduction until discontinuation of the anti-pain drugs, and progressive increase of the hemoglobin levels. At follow up of 15 months there was no evidence of active disease and the ECOG Performance Status was 0 (zero).

**Conclusion:**

The treatment was effective and feasible. Considering these excellent results, further studies about concomitant tomotherapy with Cetuximab for advanced/inoperable SCC of the skin are needed.

## Background

Cutaneous basal cell (BCC) and squamous cell carcinoma (SCC), are the most common cancer in United States [1]. SCC is the second most frequent skin tumor [2]. More than 3.000.000 new cases of SCC are diagnosed worldwide every year [3]. Incidence of SCC has risen significantly due to increased sun exposure and number of immunodeficient patients [1,4,5]. If not radically excised, SCC become invasive with tissue destruction and involvement of lymph nodes, soft tissues, cartilages, and bones. Metastatic diffusion is a rare phenomenon [1]. Generally, locoregional surgery is curative and systemic therapy is not necessary [1]. Mohs micrographic surgery is an option to be taken in account. Radiation therapy (RT) is a therapeutic option in advanced, unresectable SCC [1,6-8]. For advanced disease chemotherapy has often palliative indication [9]. Currently, the great evolution of technology has allowed RT to increase the compliance of the treatment administration and, at the same time, the reduction of the dose to the surrounding normal tissue allowing an increase of the dose to the tumor. Helical Tomotherapy (HT) delivery represents a very important step in radiotherapic technical innovation allowing improvement of dose conformation, uniformity and normal tissues sparing. Cutaneous SCC is characterized by high Epidermal growth factor receptor (EGFR) expression with low frequency of RAS mutations. These acquisitions support the potential efficacy of EGFR-target therapies. Several published data demonstrate that Cetuximab is an emerging alternative treatment for unresectable cutaneous SCC [6,10-14].

The treatment of the tumor and the maximal preservation of function are important aim in the management of cutaneous SCC. Development of Skin Cancer Index (SCI) showed that healing, cosmetic and self-image, emotional states such as anxiety and frustration were concerns greater than physical handicaps [4,15].

We evaluated the activity and toxicity profile of a new RT technology, HT, concomitant with Cetuximab, followed by Cetuximab as single agent therapy in a patient affected by unresectable, locally advanced SCC of the skin. There are limited published data available for concomitant treatment of Cetuximab and HT in advanced cutaneous SCC.

## Case presentation

On March 2012 we visited a 45-year-old Caucasian woman with a very extensive, untreated G1 cutaneous SCC infiltrating widely the lumbar region until bone. A physical examination showed a locally advanced lesions with necrotic and ulcerated areas involving the entire lumbar area until sacrum and buttocks. There was also a superinfection due to Pseudomonas Aeruginosa resistant to antibiotics. The patient referred functional limitation in movement, difficulty in walking, pain, and consequent serious relationship problems with a worse status of quality of life. The patient had G3 anemia due to the bleeding lesion, and performed red blood cells transfusion before to start the treatment. ECOG Performance Status was 2. A contrast-enhanced magnetic resonance (RM) of pelvis showed extensive skin thickening from the front region of the iliac spine up to posterior sacral region, involving the contralateral lumbar region. The lesion extended from the right of right iliac crest (where it is in contact with the lateral abdominal muscles and with the cortex of the iliac crest) until ipsilateral gluteal region, involving deep muscular tissue.

The patient’s case was discussed by a multidisciplinary committee involving surgeons, radiotherapists and oncologists. The patient was treated with Cetuximab at the loading dose of 400 mg/m2, followed by weekly doses of 250 mg/m2,from 28 March 2012 to 13 March 2013. HT has been performed concomitant with the Cetuximab from 28 march 2012 to 19 May 2012, at the total dose of 60 Gy (2 Gy/fx).

Patient was immobilized in the prone position without application of bolus. Planning computed tomography (CT) images were acquired through the region of interest using a 3 mm slice thickness and transferred to the contouring workstation for TomoTherapy Hi · Art System^®^. Set up was based on fiducial markers and tattoo aligned with a room laser system before treatment.

**Figure 1:**
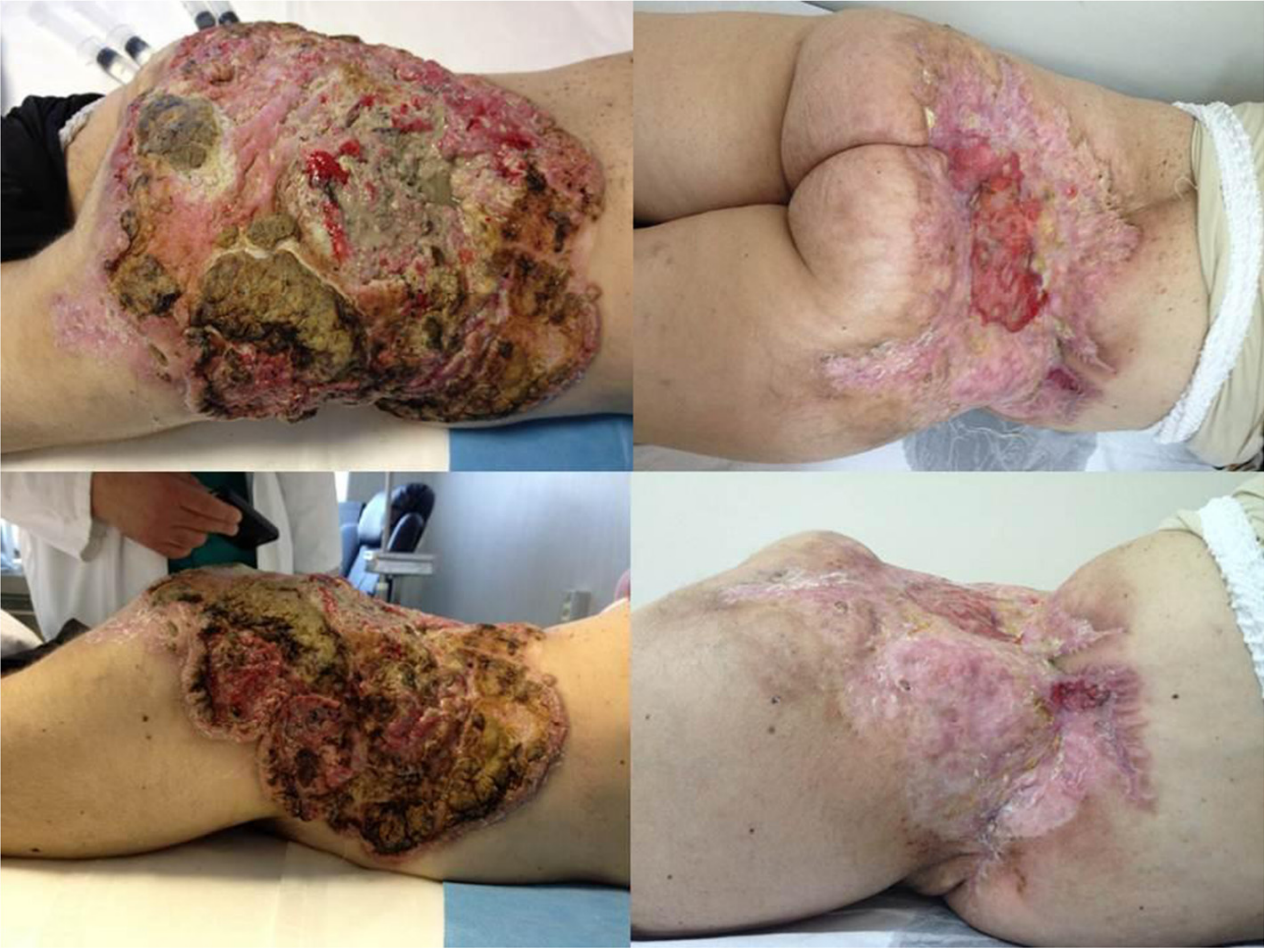
On left the lesion at baseline (March 2012) and on right the lesion at follow up (October 2013).

**Figure 2:**
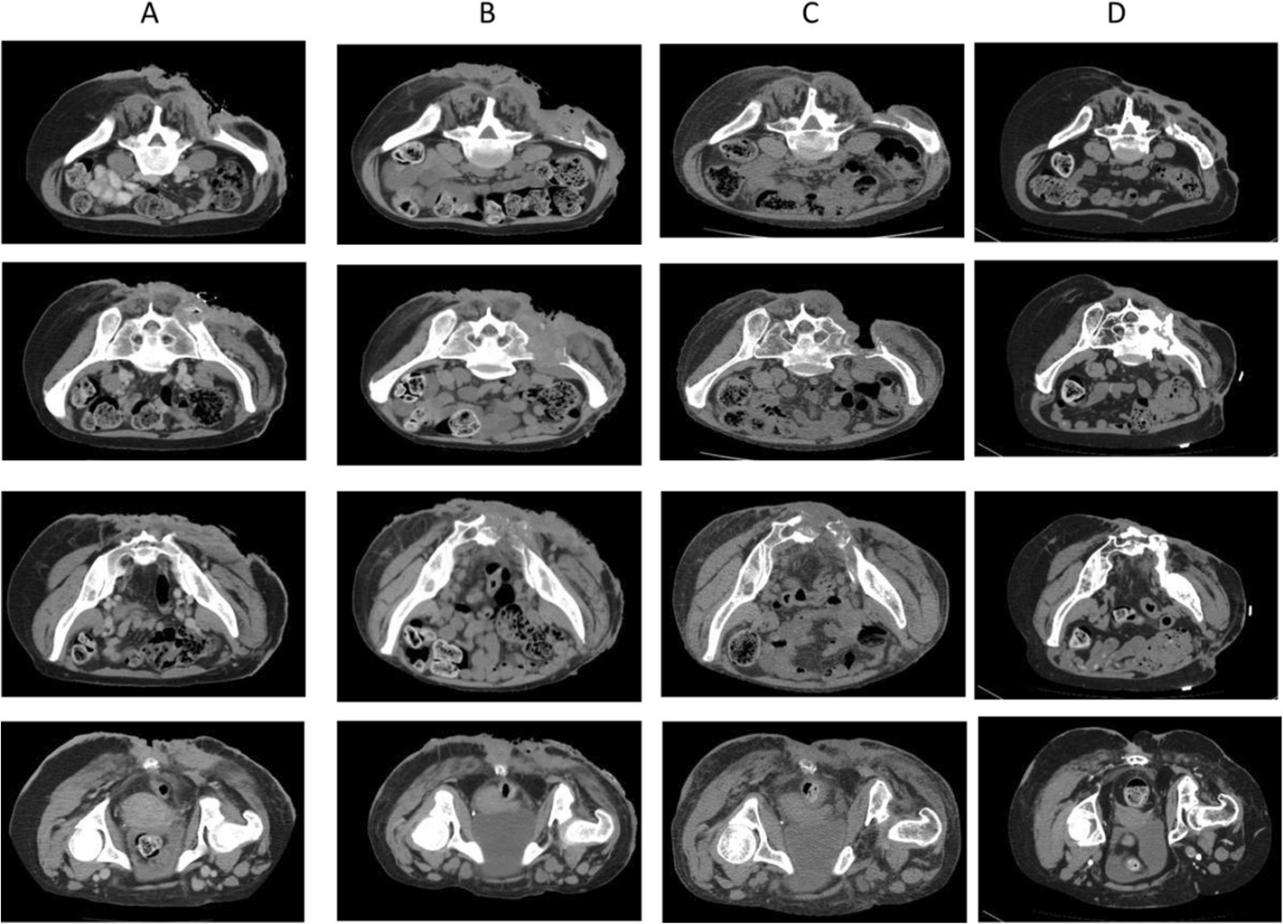
TC imaging: A baseline TC (December 2011); B simulation TC (March 2012); C follow up (June 2013); D last follow up (February 2014).

The gross target volume (GTV) was considered as right gluteal region outlined based on the depth of involvement and the extent of disease. Clinical target volume (CTV) was created adding a margin of 3 mm at GTV. Doses to organs at risk (rectum, bladder, right and left femur, right and left kidney, pubis, bowel) were defined in accordance with dose constraints. Each therapy session was preceded by a MVCT in order to ensure the correct repositioning of the patient and to adjust the distribution of the irradiation dose according with the evolution of the tumor during therapy. The accepted tolerance at our institution was 5 mm. After a new CT scan for contouring, a new treatment plan was elaborated at 30 Gy according with volumetric reduction of lesion. The time of delivery (around 10 minutes for fractions) were well tolerated by the patients. At the end of the combined radio target therapy, Cetuximab treatment continued for the following 10 months.The patient achieved complete response, confirmed at imaging evaluation, and the lesion reduced until disappear even after the suspension of the treatment (Figures 1 and 2). Toxicity resulted in G1 cutaneous rash and G2 toxicity to the nails according to typical toxicity profile of the anti-EGFR therapies, appeared 5 months after treatment. One month after therapy the Pseudomonas Aeruginosa superinfection totally disappeared as the G3 anemia.

Quality of life resulted significantly, dramatically improved with progressive reduction until discontinuation of anti-pain drugs. At follow up of 15 months there was no evidence of active disease, moreover she was able to get back a normal social life. Actually the ECOG Performance Status is 0 (zero).

## Discussion

Surgery is the standard treatment of localized cutaneous SCC [1]. Although Mohs surgery remains the standard treatment for locally advanced SCC there is no safe and effective non-surgical treatment for unresectable SCC need [6]. Traditional techniques are supported by old studies and there are few data from prospective trials. According with NCCN guidelines, radiotherapy in the management of skin cancers have indication 2A [1]. Two meta-analyses reported 10% of 5-year recurrence rates after RT for the treatment of SCC [16,17]. RT is considered the primary treatment of choice when surgical excision may result infunctional or with aesthetic compromission or for extensive unresectable tumors [1,18-20]. RT achieves excellent cosmetic results. It can be delivered using orthovoltage radiation (for superficial lesions), megavoltage electron beam technology (for deeper tissue) or brachytherapy (delivering radiation directly at the lesion) [18]. IMRT is a special technique that warranted to ensure adequate surface dose to the target area sparing normal tissue [1]. HT is an innovative treatment unit dedicated to IMRT and IGRT that consent a combination between an helical CT scanner and a conventional linear accelerator (LINAC). The RT is delivered in helical modality. In fact the 6 MV LINAC is assembled in a circular gantry similar to a CT scanner and rotates in synchrony with couch movement creating a helical trajectory. HT consent to acquire MVCT scans before to deliver RT [21]. Due to this delivery system of “record and verify”, HT allows to generate very complex dose distributions with high doses to the entire tumor volume, sparing normal healthy tissue at the same time. For advanced disease chemotherapy has often only a palliative indication. Sadek et al. [9] demonstrated that neoadjuvant chemotherapy with cisplatin, 5-fluorouracil and bleomycin followed by surgery can be curative (30% of complete remission and 54% of partial remission). The advent of systemic therapy targeted against the signaling pathway provided a new important option for patients with advanced disease unfit for local therapy [1]. EGFR plays a crucial role in signal-transduction pathways that regulate key cellular functions. Cetuximab is a recombinant monoclonal antibody that competitively inhibits EGFR. Cetuximab specifically binds to the extracellular domain of EGFR inducing an internalization of the ligand--receptor complex and subsequent down-regulation of intracellular signals [22,23]. Maubec et al. evaluated in a phase II trial the effects of Cetuximab as first line single agent in chemotherapy-naive unresectable SCC. In this phse II trial cetuximab achieved 69% DCR and 28% RR [13]. Recently, Preneau et al. [14] confirm the potential interest of cetuximab to treat unresectable advanced SCC alone or combined with carboplatin based chemotherapy or with radiotherapy.

Preclinical data on human tumor xenografts in nude mice indicated that Cetuximab in combination with radiotherapy improves local tumor control [24-26]. The radiosensitizing effect of cetuximab is probably due to regulation of cell cycle progression [27], blockage of radiation-induced EGFR transport into the nucleus, and interference with DNA repair mechanisms [28,29]. Moreover in head and neck squamous carcinomas, the combination of radiotherapy plus cetuximab is widely used in unresectable tumors.

Several studies evaluated the quality of life (QOL) perceived by the patient before and after surgical treatment [4]. Various indicators such as Skin Cancer Index (SCI) evaluating domains of emotion, social factors, and appearance [30] and the Dermatology Life Quality Index (DLQI) [31] or Skindex, reporting subscores on symptoms, emotional effects, and effects on functioning have been used [32]. In our case the combined radio-target treatment allowed the patient to achieve a significant improvement in QoL for the disappearance of the symptoms, but also and especially for the opportunity to have a social life again.

## Conclusion

Only patients with locally advanced uresectable cutaneous SCC should be considered for radiation therapy with or without target therapy. Advanced radiation therapy techniques offer greater dose escalation to tumor volumes while decreasing patient long term toxicity. HT is an effective option as radical treatment or in association with chemotherapy. Early and late toxicity and cosmetic results are acceptable. The patient will continue to be assessed in order to observe any recurrences. Considering these excellent results, further studies about concomitant Tomotherapy with Cetuximab for advanced/inoperable SCC of the skin are needed.

## Consent

Written informed consent was obtained from the patient for publication of this Case report and any accompanying images. A copy of the written consent is available for review by the Editor of this journal.

## Abbreviations

SCC: Cutaneous squamous cell carcinoma; BCC: Cutaneous basal cell; RT: Radiation therapy; HT: Helical tomotherapy; SCI: Skin cancer index; EGFR: Epidermal growth factor receptor; RM: Magnetic resonance; IMRT: Intensity-modulated radiotherapy; IGRT: Image guided radiotherapy; CT: Computed tomography; GTV: Gross target volume; CTV: Clinical target volume; LINAC: Linear accelerator.

## Competing interests

The authors declare that they have no competing interests.

## Authors’ contributions

SF, FMG, AMG, RDF conception and design, analysis and interpretation of data. FMG, AMG, DT, MM, FC acquisition of data, critical revision of important intellectual content. SF, FMG, VB drafting the manuscript and elaboration of figure. PA, PM critical revision of important intellectual content. All authors have given final approval of the version to be published.

## Pre-publication history

The pre-publication history for this paper can be accessed here:

http://www.biomedcentral.com/1471-5945/14/15/prepub
